# Galunisertib inhibits glioma vasculogenic mimicry formation induced by astrocytes

**DOI:** 10.1038/srep23056

**Published:** 2016-03-15

**Authors:** Chao Zhang, Wenliang Chen, Xin Zhang, Bin Huang, Aanjing Chen, Ying He, Jian Wang, Xingang Li

**Affiliations:** 1 Department of Neurosurgery, Qilu Hospital, Shandong University, 107 Wenhuaxi Road, Jinan, China; 2 Brain Science Research Institute, Shandong University, 44 Wenhuaxi Road, Jinan, China; 3 Institute of Basic Medical Sciences and Key Laboratory of Cardiovascular Proteomics of Shandong Province, Qilu Hospital, Shandong University, 44 Wenhuaxi Road, Jinan, China; 4 Department of Biomedicine, University of Bergen, Jonas Lies Vei 91, 5009-Bergen, Norway

## Abstract

Gliomas are among the most lethal primary brain tumors found in humans. In high-grade gliomas, vasculogenic mimicry is often detected and has been correlated with prognosis, thus suggesting its potential as a therapeutic target. Vasculogenic mimicry mainly forms vascular-like channels independent of endothelial cells; however, little is known about the relationship between astrocytes and vasculogenic mimicry. In our study, we demonstrated that the presence of astrocytes promoted vasculogenic mimicry. With suspension microarray technology and *in vitro* tube formation assays, we identified that astrocytes relied on TGF-β1 to enhance vasculogenic mimicry. We also found that vasculogenic mimicry was inhibited by galunisertib, a promising TGF-β1 inhibitor currently being studied in an ongoing trial in glioma patients. The inhibition was partially attributed to a decrease in autophagy after galunisertib treatment. Moreover, we observed a decrease in VE-cadherin and smooth muscle actin-α expression, as well as down-regulation of Akt and Flk phosphorylation in galunisertib-treated glioma cells. By comparing tumor weight and volume in a xenograft model, we acquired promising results to support our theory. This study expands our understanding of the role of astrocytes in gliomas and demonstrates that galunisertib inhibits glioma vasculogenic mimicry induced by astrocytes.

Gliomas are the most lethal intracranial tumors due to their high capacity of proliferation and invasion into healthy brain tissue, which preclude complete surgical resection^1^. As glioma invasion and proliferation rely on angiogenesis^2^, the potential of anti-angiogenic therapy to inhibit glioma progression has been investigated^3^. However, recent studies showed that although anti-angiogenic therapy might delay tumor progression, it failed to prolong long-term survival^4^,^5^. What is worse, some evidence suggests that anti-angiogenic therapy might elevate the risk of tumor adaptation and invasion in hypoxic and ischemic environments^6^,^7^. First introduced by Maniotis *et al.* in 1999^8^, vasculogenic mimicry is characterized by tumor cells forming tubular structures that transport erythrocytes and plasma in order to nourish tumors, independent of endothelial blood vessels. These structures have also been found in other types of tumors including breast^9^, lung^10^, and ovarian^11^. As in gliomas, vasculogenic mimicry was also detected predominantly in high-grade medulloblastomas and there was a significant association between vasculogenic mimicry and medulloblastoma grade^12^. Researchers have suggested that vasculogenic mimicry enabled gliomas to survive in hypoxic and ischemic environments^13^, and thus explain the limitations of anti-angiogenic therapy^14^. Apart from anti-angiogenic therapy, anti-vasculogenic mimicry therapy should be considered for treatment of gliomas^15^. However, investigation of the mechanisms of vasculogenic mimicry stimulation and inhibition are required.

In the brain, hypoxia, ischemia, and the presence of glioma cells cause chronic inflammation resulting in recruitment of cell types such as astrocytes and microglia; reactive astrocytes often in turn surround gliomas and brain metastases^16^,^17^. Although the physiological function of astrocytes is to protect neurons^18^, they also seem to enhance tumor cell survival signaling pathways^19^ and increase their resistance to chemotherapy. In addition, reactive astrocytes express numerous genes that support tumor cell survival in a paracrine manner^20^, where hypertrophic astrocytes secrete chemokines that promote tumor survival and invasion^21^,^22^. Specifically, reactive astrocytes have been shown to secrete TGF-β, which increases tumor cell proliferation, as well as connective tissue growth factor and metalloproteases, facilitating glioma invasion^23^.

Galunisertib (LY2157299), a selective ATP-mimetic inhibitor of TGF-βRI, is one of the few TGF-β pathway inhibitors currently under clinical investigation in glioma patients^24^. In recent clinical trials^24^,^25^, galunisertib improved glioma prognosis. However, *in vitro* experiments have not been able to explain its mechanism of action and the relationship between galunisertib and astrocytes has not yet been reported. Our research uncovers the effects of galunisertib on gliomas, particularly on vasculogenic mimicry. Our results also show the potential influence of galunisertib on autophagy, an important process responsible for tumor metabolism and invasion. These findings suggest a new strategy for discovery of novel vasculogenic mimicry therapeutics.

## Results

### Human astrocytes promote vasculogenic mimicry in glioma cell line A172

**Figure 1:**
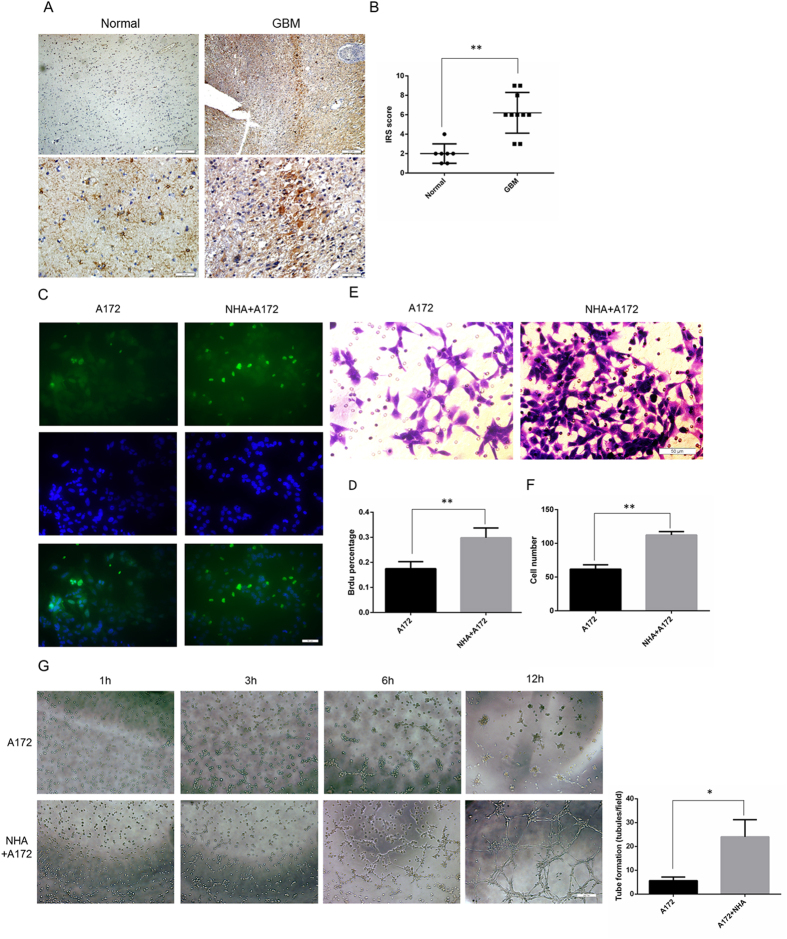
Human astrocytes promoted vasculogenic mimicry in glioma cell line A172. (**A**) Representative images and (**B**) immunoreactive scoring of GFAP staining of paraffin-embedded specimens obtained from decompression surgery and glioma patients. Astrocytes were more abundant in gliomas than in normal brain tissue. (**C**) Representative images and (**D**) quantification of the Brdu proliferation assay. More Brdu^+^ cells were observed in the NHA/A172 co-culture cells than in the A172 cells alone. Green represents Brdu^+^. Blue represents DAPI. (**E**) Representative images and (**F**) quantification of the Transwell migration assay. More cells migrated through Matrigel in the NHA/A172 co-culture than in A172 cells alone. (**G**) Representative images and quantification of *in vitro* vasculogenic mimicry tube formation assay. NHA/A172 co-culture induced more tube formation than A172 cells alone. Mean ± SEM of three independent experiments; *P < 0.05; **P < 0.01. Scale bars of the upper line in Fig. 1A represent 200 μm, the others represent 50 μm.

Astrocytes, which comprise approximately 50% of the cells in the brain^26^, play a vital role in glioma proliferation, invasion, and angiogenesis^16^,^19^. As shown in Fig. 1A, astrocytes stained with glial fibrillary acidic protein (GFAP) were more abundant in glioblastomas (GBM) than in normal brain tissue. The astrocytes in the GBM clustered, forming a border encompassing the tumors, which differs from the grid distribution of normal brain tissue. Quantification and immunoreactive scoring (IRS) reflected these observations (Fig. 1B, one-way ANOVA GBM 6.20 ± 0.66, N = 7 *vs.* normal 2.00 ± 0.37, N = 10, *P* = 0.0002).

To study the effect to astrocytes on gliomas, we established a co-culture model using normal human astrocytes (NHA) and a glioma cell line (A172) cultured in Transwell chambers. A successful model of reactive astrocytes should promote glioma proliferation and invasion^19^,^21^, which we measured using Brdu proliferation assays and Transwell migration assays. Significantly more Brdu^+^ cells were detected in the NHA/A172 co-culture system than in the A172 cells cultured alone (Fig. 1C,D, A172 cells alone 17.26 ± 1.29%, N = 6 *vs*. NHA/A172 co-culture 30.81 ± 1.03%, N = 6, *P* = 0.0018). Cell migration was increased in the co-culture system compared to A172 cells alone (Fig. 1E,F, A172 alone 61.67 ± 6.56, N = 6 *vs*. NHA/A172 co-culture 112.31 ± 5.04, N = 6, *P* = 0.0036). Thus, astrocytes in the co-culture model were active.

Next we studied the role of astrocytes in vasculogenic mimicry. The NHA/A172 co-culture group demonstrated more rapid tube formation (Fig. 1G, tube length per unit time: A172 alone 5.66 ± 0.8819, N = 3 *vs*. NHA/A172 co-culture 24.00 ± 4.16, N = 3, *P* = 0.0125). To our knowledge, this is the first finding to suggest that astrocytes may enhance glioma proliferation and invasion by accelerating vasculogenic mimicry.

### Astrocyte-induced vasculogenic mimicry required TGF-β1 secretion and was inhibited by galunisertib

**Figure 2:**
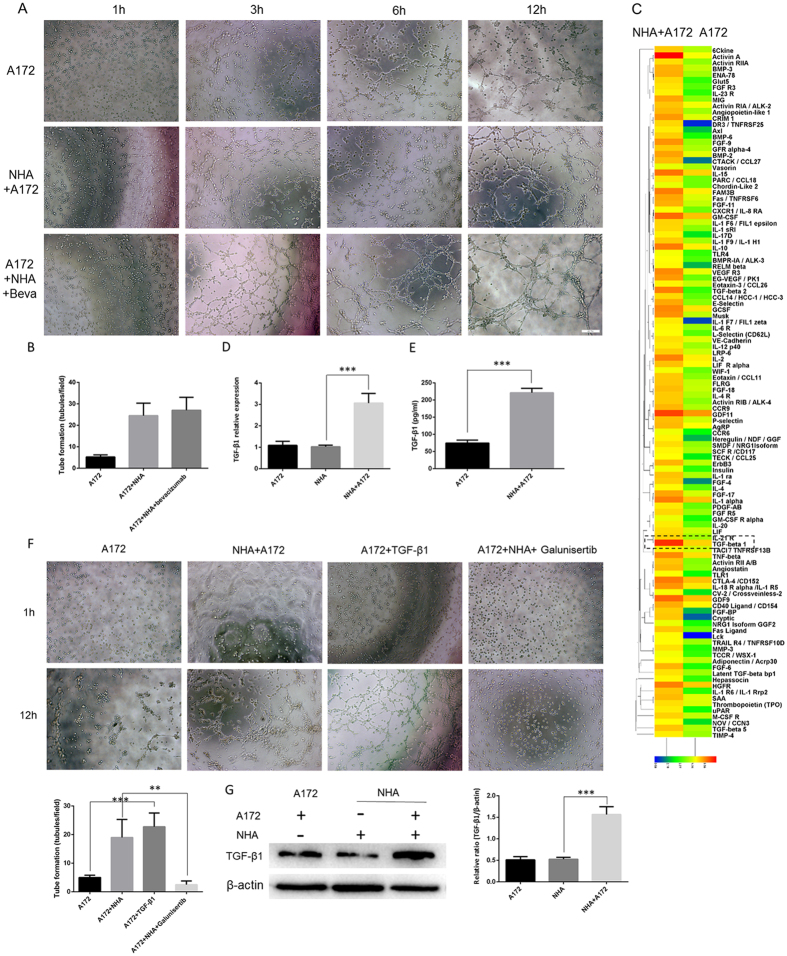
Astrocyte-induced vasculogenic mimicry required TGF-β1 secretion and was inhibited by galunisertib. (**A**) Representative images and (**B**) quantification of *in vitro* vasculogenic mimicry tube formation assay. No significant differences were found between bevacizumab-treated NHA/A172 cells (Beva, 10 μg/mL) and the control NHA/A172 cells. (**C**) Suspension microarray analysis of the supernatant from NHA/A172 co-cultures and A172 cells alone. TGF-β1 was elevated in the NHA/A172 culture media compared to the A172 alone media. (**D**) qRT-PCR analysis of TGF-β1 mRNA expression in A172 alone, NHA alone and NHA/A172 co-culture and (**E**) ELISA analysis of TGF-β1 concentration in the supernate of A172 cells with or without NHA co-culture (**F**) Representative images and quantification of *in vitro* vasculogenic tube formation assay indicated that the TGF-β1-treated cells (10 ng/mL, 24 h) formed vessel-like structures similar to the NHA/A172 cells. TGF-β1 inhibitor, galunisertib, inhibited astrocyte-induced tube formation. (**G**) Quantification of immunoblotting, NHA cells was found to raise their expression level of TGF-β1 only after co-cultured with A172. Mean ± SEM of three independent experiments; **P < 0.01; ***P < 0.001. Scale bar represents 50 μm.

Previous studies have reported that vasculogenic mimicry nourishes gliomas by supporting tumor proliferation and invasion^27^ in hypoxic and ischemic environments, which is partially similar to the process of tumor angiogenesis. Thus, we explored whether the anti-angiogenesis drug bevacizumab could inhibit astrocyte-induced vasculogenic mimicry. Not surprisingly, there was no significant difference in tube formation after bevacizumab treatment (Fig. 2A,B, control NHA/A172 cells 24.50 ± 2.90, N = 4 *vs*. bevacizumab-treated NHA/A172 cells 27.00 ± 3.28, N = 4, *P* = 0.5728).

Interestingly, a recent publication indicated that tumor vasculogenic mimicry is independent of the VEGF pathway^28^. In addition, reports showed that active astrocytes secrete many tumor-related cytokines^22^,^23^, and it is possible that these cytokines contribute to vasculogenic mimicry. To screen potential target cytokines, a suspension array technique was employed. We found that TGF-β1 was highly expressed in the supernatant of NHA/A172 co-culture system (Fig. 2C). Given that TGF-β1 is a multi-functional cytokine in tumor angiogenesis^29^, we hypothesize that it also plays a key role in astrocyte-induced vasculogenic mimicry which may be blocked by its inhibitor. The suspension array results were confirmed by qRT-PCR and ELISA. Both mRNA levels in two experimental NHA groups (Fig. 2D, NHA alone 1.025 ± 0.03, N = 5 *vs*. NHA/A172 co-culture 3.06 ± 0.19, N = 5, *P* < 0.0001) and protein concentration (Fig. 2E, A172 alone 74.67 ± 5.00 pg/mL, N = 3 *vs*. NHA/A172 co-culture 221.30 ± 7.422 pg/mL, N = 3, *P* < 0.0001) of TGF-β1were elevated in the NHA/A172 co-cultured cells.

Using the *in vitro* tube formation assay, we observed that A172 cells treated with TGF-β1 demonstrated elevated tube formation compared to untreated A172 cells (10 ng/mL for 24 h; Fig. 2F, untreated A172 cells 5.20 ± 0.40, N = 4 *vs*. TGF-β1-treated A172 cells 22.75 ± 2.39, N = 4, *P* = 0.0003). Importantly, galunisertib inhibited the effect above (Fig. 2F, untreated NHA/A172 cells 19.00 ± 3.16 N = 4, *vs*. galunisertib-treated NHA/A172 cells 2.50 ± 0.64, N = 4, *P* = 0.0022).

With quantification of immunoblotting, we found that NHA cells raised their expression level of TGF-β1 only after co-cultured with A172 (Fig. 2G, NHA alone 0.53 ± 0.02 N = 3 *vs* NHA/A172 1.29 ± 0.07 N = 3).

Together, our data indicated that astrocytes promoted vasculogenic mimicry tube formation, possibly through the secretion of TGF-β1. These results also show that bevacizumab, a traditional anti-angiogenesis drug, failed in preventing vasculogenic mimicry tube formation. Instead, galunisertib, a TGF-β1 inhibitor, may be an alternatively effective anti-glioma therapy.

### Galunisertib inhibited SMα and VE-cadherin expression in the NHA/A172 co-culture system via Akt and Flk pathways

**Figure 3:**
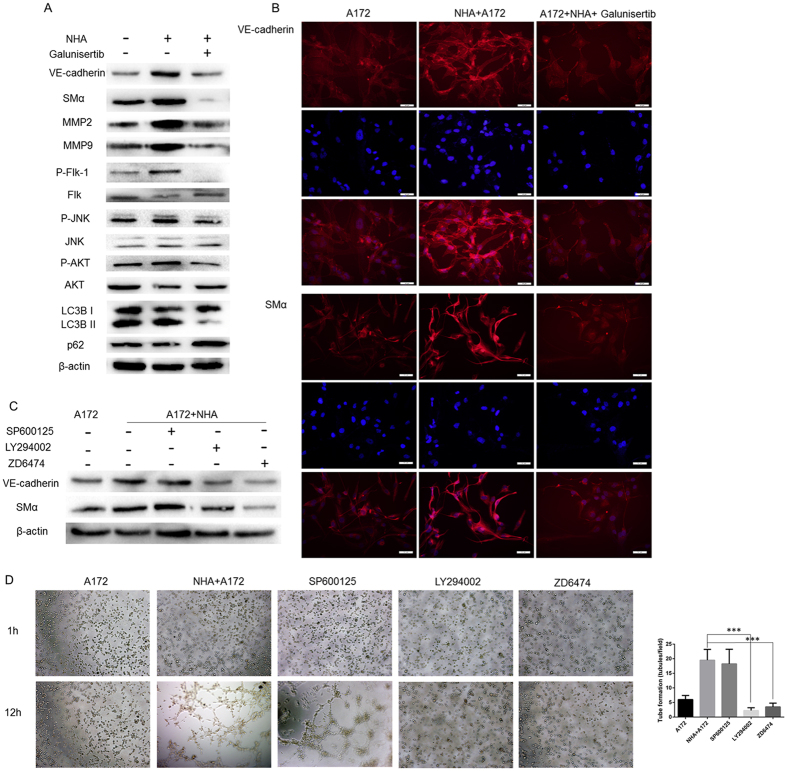
Galunisertib inhibits SMα and VE-cadherin expression in NHA/A172 co-cultured cells via Akt and Flk pathways. (**A**) Representative Western blot analysis of lysates from A172 cells alone, DMSO-treated NHA/A172 cells, and galunisertib-treated NHA/A172 cells. SMα, VE-cadherin, phosphorylated Flk, and phosphorylated Akt were elevated in the DMSO-treated NHA/A172 cells only. (**B**) Immunofluorescence of SMα (Red) and VE-cadherin (Red) combined with DAPI (blue) revealed that VM markers were elevated in DMSO-treated NHA/A172 cells but not in galunisertib-treated NHA/A172 cells. (**C**) Western blotting revealed that treatment with LY294002 or ZD6474 reduced SMα and VE-cadherin expression in cell lysates from the NHA/A172 co-culture system. (**D**) Representative images and quantification of tube formation assay. LY294002 or ZD6474 treatment reduced tube formation in NHA/A172 co-cultured cells. Mean ± SEM of three independent experiments; ***P < 0.001. Scale bar represents 50 μm.

To further examine the roles of astrocytes and galunisertib in vasculogenic mimicry and to explore the potential mechanism, we studied molecular markers previously identified by Hendrix^1^ and Seftor *et al.*^30^. Western blot analysis revealed that co-culture with astrocytes elevated SMα, VE-cadherin, matrix metalloproteinase-2 (MMP-2), and MMP-9 protein expression in A172 cells. Meanwhile, treating the NHA/A172 cells with galunisertib dramatically down-regulated expression of these markers in A172 cells (Fig. 3A). Immunofluorescence also showed elevated SMα and VE-cadherin expression in DMSO-treated NHA/A172 cells and not in galunisertib-treated NHA/A172 cells (Fig. 3B).

Francescone^28^ and Kirschmann *et al.*^27^ identified SMα and VE-cadherin as the markers of VM. Given these findings and combined with the old markers-MMP2, MMP9, we examined several signaling pathways that may have been involved in vasculogenic mimicry. We found that Akt and Flk phosphorylation in the galunisertib group varied dramatically compared to the NHA/A172 group. In addition, LC3B expression level was also changed which would be further discussed in the following part (Fig. 3A). To identify the precise role of these signaling pathways, we treated A172 and NHA/A172 cells with LY294002 (a PI3K-Akt pathway inhibitor), ZD6474 (a Flk pathway inhibitor), and SP600125 (a JNK pathway inhibitor). Using Western Blot, we found decreased SMα and VE-cadherin expression after LY294002 or ZD6474 treatment with A172 alone and A172/NHA control groups (Fig. 3C). Similarly, an *in vitro* tube formation assay demonstrated that inhibiting Akt and Flk pathways blocked astrocyte-induced vasculogenic mimicry tube formation (Fig. 3D, A172 cells alone 6.00 ± 0.711 N = 4, untreated NHA/A172 cells 19.50 ± 1.84, N = 4 *vs*. LY294002-treated cells 2.250 ± 0.4787, N = 4, *P* = 0.0001, and *vs*. ZD6474-treated cells 3.50 ± 0.64, N = 4, *P* = 0.0002). Inhibiting the JNK pathway did not significantly change SMα and VE-cadherin expression or tube formation (Fig. 3D, SP600125-treated cells 18.25 ± 2.496, N = 4, *P* = 0.7013). Therefore, our data demonstrated that galunisertib inhibited SMα and VE-cadherin expression likely through inhibition of the Akt and Flk signaling pathways.

### Galunisertib impaired glioma autophagy, which in turn inhibited vasculogenic mimicry

**Figure 4:**
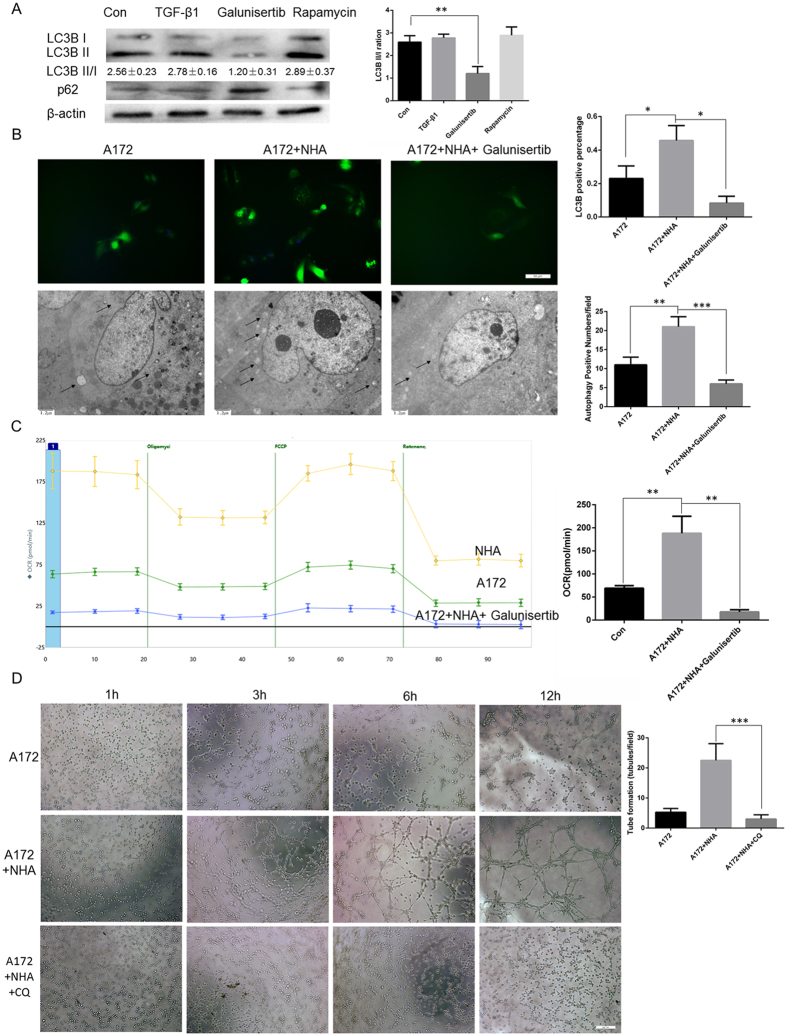
Galunisertib impaired glioma autophagy, which in turn inhibited vasculogenic mimicry. (**A**) Western blotting showed that galunisertib suppressed LC3B, an autophagy marker, in A172 cells. (**B**) Representative images and quantification of immunofluorescence (top, scale bar 50 μm) and transmission electron microscopy (bottom, scale bar 1.2 μm). The number of punctate LC3 structures and of autophagosomes (arrows) were increased in NHA/A172 cells and not in galunisertib-treated cells. (**C**) Oxygen consumption rate (OCR) was measured by XFe24 with a mito stress kit. Galunisertib lowered metabolic rate. (**D**) Representative images and quantification of *in vitro* tube formation assay. Scale bar represents 50 μm. Autophagy inhibition with chloroquine (CQ) inhibited tube formation. Mean ± SEM of three independent experiments; *P < 0.05; **P < 0.01; ***P < 0.001.

As TGF-β1 has been reported to be associated with autophagy^31^ and autophagy with vasculogenic mimicry^32^, we investigated whether galunisertib (TGF-β inhibitor) inhibits vasculogenic mimicry by regulating glioma autophagy. Immunofluorescence revealed that galunisertib did suppress glioma autophagy (Fig. 4A). In addition, the number of punctate LC3B structures, a standard marker of autophagy, was significantly decreased after galunisertib treatment (Fig. 4B, LC3B^+^: A172 alone 0.23 ± 0.043%, N = 4 *vs*. NHA/A172 co-culture 0.45 ± 0.05%, N = 4, *P* = 0.0286; galunisertib-treated cells 0.083 ± 0.02% *vs*. NHA/A172 co-culture 0.45 ± 0.05, N = 4, P = 0.0413). Transmission electron microscopy (TEM), the gold standard technique for identification of double-membrane autophagosomes, was then conducted. We found that galunisertib-treated cells had fewer autophagosomes (Fig. 4B, autophagosomes/field: A172/NHA co-culture 21.00 ± 1.52, N = 3 *vs*. A172 alone 11.00 ± 1.15, N = 3, *P* = 0.0064; and vs galunisertib-treated cells 6.000 ± 0.57, N = 3, *P* = 0.0008).

Previous reports have shown that autophagy is involved in glioma metabolism, which provides energy and substrates for vasculogenic mimicry^33^. We thus examined the effect of galunisertib on glioma mitochondrial metabolism using seahorse XFe24. Results showed that NHA/A172 co-cultured cells had an elevated oxygen consumption rate (OCR) indicating more active ATP production and that galunisertib significantly lowered OCR (Fig. 4C, NHA/A172 188.1 ± 21.33, N = 3 *vs*. A172 69.03 ± 3.28, N = 3, *P* = 0.0053; galunisertib-treated NHA/A172 17.66 ± 2.74, N = 3 *vs*. NHA/A172 188.1 ± 21.33, N = 3, *P* = 0.0014). Recognizing the role of autophagy in vasculogenic mimicry, we hypothesized that autophagy inhibitors, such as chloroquine (CQ), may inhibit vasculogenic mimicry. As a result, the tube formation assay revealed that CQ mirrored the effect of galunisertib (Fig. 4D, CQ-treated NHA/A172 cells 3.00 ± 0.70, N = 4 *vs*. NHA/A172 cells 22.50 ± 2.78, N = 4, *P* = 0.0009).

In summary, galunisertib suppressed autophagy in gliomas, which lowered metabolic rate and influenced vasculogenic mimicry. This provides an additional mechanism explaining how galunisertib inhibits astrocyte-induced vasculogenic mimicry.

### Galunisertib inhibited tumor growth and vasculogenic mimicry in a xenograft tumor model

**Figure 5:**
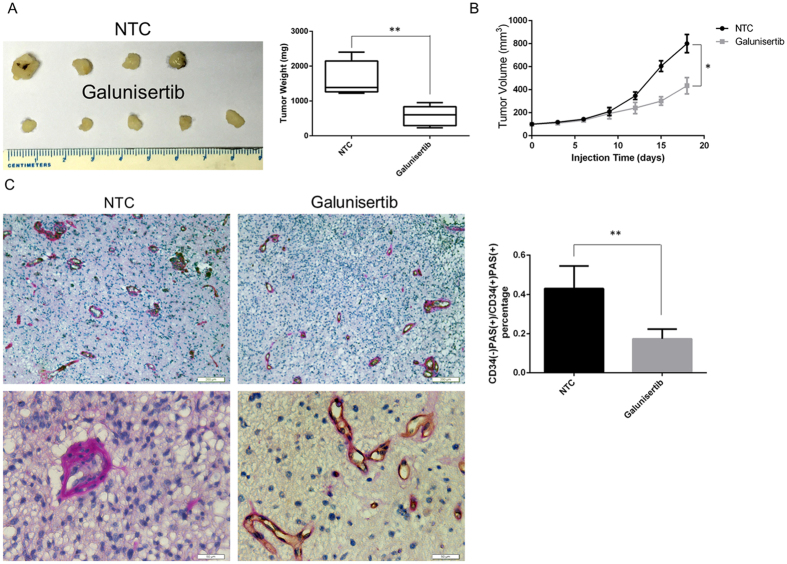
Galunisertib inhibited tumor growth and vasculogenic mimicry in a xenograft tumor model. (**A, B**) Mice were sacrificed 21 days after the injection of normal tumor cells (NTC) with or without galunisertib treatment. Tumor weight (**A**) and volume (**B**) were measured. (**C**) Immunohistochemical CD34 and PAS staining of tumors sections isolated from galunisertib-treated and PBS-treated (20% DMSO) mice. Scale bars represent 200 μm (top) and 50 μm (bottom), respectively. The percentage of PAS^−^CD34^+^/PAS^+^CD34^+^ cells decreased in the galunisertib-treated xenografts, representing reduced tumor vasculogenic mimicry *in vivo*. Mean ± SEM of three independent experiments; *P < 0.05; **P < 0.01.

To verify the *in vitro* findings, we established a xenograft tumor model using the A172 cell line. Mice were randomly assigned to two groups. No major side effects was observed throughout the study. As shown in Fig. 5A,B, galunisertib significantly reduced average tumor weight and volume compared to controls treated with normal tumor cells (NTC) alone (weight: NTC group 1598 ± 270.3 mg, N = 4 *vs*. galunisertib-treated group 570.0 ± 128.8 mg, N = 5, *P* = 0.0078; volume: NTC group 584.8 ± 26.95 mm^3^, N = 4 *vs*. galunisertib-treated group 93.00 ± 7.74 mm^3^, N = 5, *P* = 0.0321). Importantly, we observed a pronounced decrease in tumor vasculogenic mimicry in the galunisertib-treated xenografts noted by the percentage of PAS^-^CD34^+^/PAS^+^CD34^+^ cells identified using immunohistochemistry (Fig. 5C, NTC group 0.43 ± 0.07, N = 5 *vs*. galunisertib-treated group 0.17 ± 0.03, N = 4, *P* = 0.0085). Consistent with the *in vitro* observations, these data showed that galunisertib inhibited vasculogenic mimicry tube formation and proliferation in a xenograft tumor model.

## Discussion

Reactive astrocytes constitute a major component of brain tumor microenvironment^26^,^34^; however, the majority of previous studies have focused on the gliomas themselves. Thanks to research by CC Naus, NA Charles, and others^14^,^16^,^19^,^35^, we have now identified astrocytes as an important multifunctional factor that enhances expression of survival genes in gliomas, regulates tumor cell invasion, and supports tumor resistance to the chemotherapy. However, the function of astrocytes in tumor formation is not well understood. To our knowledge, this is the first study to use an astrocytes/glioma co-culture system in defining vasculogenic mimicry. An emerging concern in glioma research, vasculogenic mimicry, is now used to evaluate tumor invasion and prognosis for glioma patients^11^,^27^. However, the mechanism of vasculogenic mimicry remains unclear^27^. TGF-β1 is one of the inducers of vasculogenic mimicry, but the origin of TGF-β1 in the glioma microenvironment was previously unknown. Astrocytes usually regulate glioma oncogenesis through paracrine manners^36^. Here we provide evidence that astrocytes secreted TGF-β1 to induce vasculogenic mimicry in GBM cells and that inhibition of TGF-β1 blocked astrocyte-induced vasculogenic mimicry.

Galunisertib, a novel anti-cancer therapeutic, is the only inhibitor of TGF-β pathway under clinical investigation. It is currently being studied in patients with hepatocellular carcinoma (NCT01246986)^37^ and has demonstrated improvement of survival time in triple-negative breast cancer^38^ and gliomas^39^. Compared to the traditional anti-angiogenesis therapy bevacizumab, the efficacy of galunisertib is promising according to recent clinical studies. However, the reason why TGF-β inhibition turns out more effective than conventional anti-angiogenesis therapy is unclear. Some researchers have attributed the failure of bevacizumab to spontaneous vasculogenic mimicry^12^,^13^,^15^,^28^ and others have suggested that astrocytes support glioma resistance to chemotherapy^26^,^35^. Perhaps, then, bevacizumab is unable to block astrocyte-induced vasculogenic mimicry.

SMα and VE-cadherin have been identified as key markers for vasculogenic mimicry^1^. In normal blood vessels, VE-cadherin adheres adjacent vascular endothelial cells to each other in order to ensure an impermeable vessel wall, while SMα allows vascular smooth muscle cells to cause vessel dilation and constriction. MMP-2 and MMP-9 are enzymes that degrade the extracellular matrix to allow for new vessel formation. These four factors are also related to epithelial-to-mesenchymal transition (EMT), a key process that enables tumor cell invasion and metastasis. As TGF-β1 has been demonstrated to be a forceful inducer of EMT^40^, there may be an innate association between EMT and vasculogenic mimicry. If so, the effect of galunisertib may extend to EMT. This interesting question warrants further investigation.

A study by Francescone *et al.* demonstrated that glioma vasculogenic mimicry relied on Flk pathway and was independent of VEGF^41^. However, VEGFR2 was reported to play a key role in vasculogenic mimicry^42^. Hence, the process and mechanism of vasculogenic mimicry is complex and poorly understood. In our study, we demonstrated that autophagy may participate in the process of vasculogenic mimicry.

An association between autophagy and vasculogenic mimicry was also reported by previous studies and demonstrated in our experiments. Evidence also suggests that purines, such as ATP, play a significant role in cancer progression and that mitochondria function is essential for vasculogenic mimicry^43^,^44^. After demonstrating the efficacy of galunisertib to decrease glioma autophagy, we hypothesized that the mechanism might be as follows: galunisertib down regulates autophagy, which in turn decreases ATP and causes mitochondrial damage, ultimately limiting glioma proliferation and invasion. Further study is necessary to confirm this hypothesis. In addition, given that CQ also successfully inhibited vasculogenic mimicry, the exact role of autophagy in vasculogenic mimicry requires more discussion.

In summary, our study demonstrated that astrocytes induced glioma vasculogenic mimicry in a co-culture system through secretion of TGF-β1. Galunisertib blocked this process at least partially via inhibition of autophagy. Encouragingly, restrained vasculogenic mimicry provided an explanation for the clinical efficacy of galunisertib. Thus, our study sheds new mechanistic insight into vasculogenic mimicry and offers a novel therapeutic target for treatment of glioma patients.

## Materials and Methods

### Cell culture and reagents

The high-grade human glioma cell line A172 was obtained from the American Type Culture Collection (Manassas, VA, USA) and the normal human astrocytes (NHA) were purchased from Lonza (Walkersville, MD, USA). Both were used for *in vitro* experiments. Tumor cells were maintained as monolayer cultures in Dulbecco’s Modified Eagle’s Medium (DMEM; GIBCO) supplemented with 5% fetal calf serum, 100 units/mL of penicillin, and 100 μg/mL of streptomycin in humidified air with 5% CO_2_ at 37 °C. NHA were cultured in Astrocyte Medium BulletKit (Lonza) according to the manufacturer’s instructions and stained positive for the marker glial fibrillar acidic protein (GFAP). As indicated, cells were treated with TGF-β1 (10 ng/mL; PeproTech) and/or the small molecule TGF-β receptor inhibitor galunisertib (LY2157299, 10 μM; Selleck), chloroquine (25 μM; Selleck), or rapamycin (20 μM; Selleck).

### Transwell co-culture and invasion assay

Invasion potential was determined on collagen-coated Transwell assay inserts with 8 μm pore size (Corning). The A172 cells were trypsinized and 150 μL of 2.5 × 10^4^ cells were added to each Transwell. NHA (1 × 10^4^ cells) were plated in the lower wells. In the co-culture Transwell (0.2 μm pore size) system however, A172 cells were trypsinized and 150 μL of 2.5 × 10^4^ cells were added to the lower wells, while 1 × 10^4^ NHA were plated in the upper wells. Culture media was same as above.

Transwell insert membranes were fixed with 75% methanol/25% acetic acid for 20 minutes, stained with 0.25% eosin in 45% methanol/10% acetic acid, and washed with demi water. Membranes were subsequently cut out and mounted on microscopy slides for quantification. Representative pictures of the membranes with cells were acquired at 40× magnification with Olympus BX61 microscope. The total number of cells in 10 individual fields per membrane were counted.

### Suspension microarray assay

To screen for vasculogenic mimicry activators, we conducted a suspension microarray assay (RayBioteh LI507) following the manufacturer’s instructions. Supernatants were collected after A172 was co-cultured with NHA in DMEM with 20% serum in 24-well culture plates at 2.5 × 10^4^ cells/well for 24 h.

### Brdu proliferation assay

Glioblastoma (GBM) cells were plated on lower chambers (2.5 × 10^4^ cells/well) with astrocytes on the upper chambers in 24-well Transwell plates (0.4 μm pore size) for 24 h. Cells were treated for an additional 48 h in DMEM with 20% serum. The lower chambers with the GBM cells were stained with Brdu using an Apollo detection kit (Ribobio, Inc.) according to the manufacturer’s instructions. Brdu^+^ cells were counted from at least 100 random fields under a fluorescence microscope.

### *In vitro* tube formation assay

A vasculogenic mimicry network was established as described by El Hallani *et al.*^45^. Briefly, 24-well tissue culture plates were coated with Matrigel Basement Membrane Matrix (500 μl/well, BD Bioscience), which was allowed to polymerize at 37 °C for 1 h. Cells (2.5 × 10^5 ^cells/mL) were seeded on Matrigel, and incubated without serum in 5% CO_2_ at 37 °C for 24 h. To investigate the effect of NHA on A172 vasculogenic mimicry, co-cultures preceded the tube formation assays with Transwell chambers (0.4 μm pore size). After 24 h, images were captured by an Olympus BX61 fluorescence microscope. The number of tubular structures in five randomly chosen 20× fields were quantified using an Olympus Microsystem.

### Immunohistochemistry

Formalin-fixed paraffin-embedded human glioma specimen sections were deparaffinized, rehydrated, boiled in sodium citrate buffer for antigen retrieval, and blocked for endogenous peroxidase activity. After careful immunostaining with primary monoclonal antibodies targeting GFAP (rabbit monoclonal, 1:100, Abcam), CD34 (rabbit monoclonal, 1:100, Abcam) together with PAS (Sigma Aldrich) overnight at 4 °C, sections were incubated with poly-HRP secondary antibodies for 30 min, developed with 3,3′-diaminobenzidine, and counterstained with hematoxylin. Images were captured using an Olympus IX81 microscope and analyzed using an immunoreactive scoring (IRS) model, where IRS = SI (staining intensity) × PP (percentage of positive cells).

### Immunofluorescence

NHA and A172 glioma cells (1:2) were co-cultured in 24-well culture plates for 48 h. Cells were fixed in 4% paraformaldehyde, permeabilized with 0.5% Triton X-100, and blocked with 10% normal goat serum. Cells were then incubated with primary antibodies targeting LC3B (rabbit monoclonal, 1:400, Cell Signaling), VE-cadherin (rabbit monoclonal, 1:300, Abcam), or SMα (rabbit monoclonal, 1:500, Abcam) overnight at 4 °C, followed by incubation of a secondary antibody conjugated with Dylight 594 fluorescent dyes (1:100, goat anti-rabbit IgG, Abbkine) for 1 h at 37 °C. Nuclei were counterstained with DAPI, and cells were imaged using an Olympus IX81 microscope.

### RNA isolation and quantitative real-time PCR

Total RNA of A172 cells was isolated by TRIzol, and cDNA was synthesized using a ReverTra Ace qPCR RT Kit (Toyobo) according to the manufacturer’s instructions. Then, quantitative real-time PCR was used to determine TGF-β1 mRNA expression. Primer sequences were obtained from Primer Bank as follows (forward and reverse, respectively): TGF-β1, 5′-CTA ATG GTG GAA ACC CAC AACG-3′ and 5′-TAT CGC CAG GAA TTG TTG CTG-3′, and GAPDH, 5′-GGT GGT CTC CTC TGA CTT CAA CAG-3′ and 5′-GTT GCT GTA GCC AAA TTC GTT GTG-3′. Target gene expression levels were normalized to that of GAPDH in the same reaction.

### Western blot

Total cell extracts were separated by SDS–polyacrylamide gel electrophoresis and transblotted to polyvinylidene difluoride membranes (0.22 μm, Millipore). Membranes were then incubated with rabbit anti-LC3B, P62, P-Flk, Flk, P-Akt, Akt, P-JNK, JNK, VE-cadherin, SMα, MMP-2, and MMP-9 polyclonal antibodies (1:1,000) or mouse anti-β-actin monoclonal antibodies (1:1,000) overnight at 4 °C and probed with the appropriate secondary antibodies. Bands were examined using Western Chemiluminescent HRP substrate (Millipore Corporation, Billerica, USA) and imaged using an Image Station 4000MM Pro (Carestream Health Inc., Woodbridge, MA, USA).

### Enzyme-linked immunosorbent assay

To quantify activated human TGF-β1 concentrations in the cell culture supernatants, the quantitative sandwich enzyme immunoassay technique was used with commercially available, specific immunoassay kits for human TGF-β1 (R&D Systems). The minimum detectable dose of TGF-β1 was less than 7.0 pg/mL. The assay was performed in triplicate according to the manufacturer’s instructions.

### Tumor xenograft model

Animal experiments conformed to the Animal Management Rule of the Chinese Ministry of Health (documentation 55, 2001) which is in accordance with the approved guidelines, and the experimental protocol was approved by the Animal Care and Use Committee of Shandong University. BALB/c nude (nu/nu) female mice were purchased from Vital River Laboratories. A172 cells (5 × 10^6^ cells in 50 μl of serum-free DMEM) were inoculated subcutaneously into the right axilla of 5-week-old female mice after acclimatization for 1 week. Tumor growth was measured daily with calipers. Tumor volume was calculated as (L × W^2^)/2, where L was the length in millimeters and W was the width in millimeters. When the tumors reached a mean volume of 70–150 mm^3^, animals were randomized to two groups. Ten mice were assigned to the normal tumor cell (NTC) group fed with equal volume of PBS containing 20% DMSO. The galunisertib group was fed galunisertib (75 mg/kg/d in 20% DMSO in PBS).

### XFe24 oxygen consumption analysis

Oxygen consumption rate (OCR) was measured using the XFe24 Analyzer (Seahorse Bioscience) as described by Guo *et al.*^46^ following the manufacturer’s instructions. Briefly, cells were seeded at 5 × 10^4^ cells per well in XFe24 plates in 100 mL of media and incubated for 12 h prior to baseline OCR measurements. The XF assay medium was low buffered bicarbonate-free DMEM (pH 7.4). Oligomycin, FCCP, and rotenone were then added as advised in sequence according to the XF cell mito stress test kit (Seahorse Bioscience). Results were obtained and analyzed by Wave software 2.2.0 (Seahorse Bioscience).

### Statistical analyses

All experiments were repeated at least three times. All data are presented as mean ± SEM. Statistical comparisons between means were made using Student’s t tests. Statistical significance is indicated as follows: *P < 0.05; **P < 0.01; ***P < 0.001.

## Additional Information

**How to cite this article**: Zhang, C. *et al.* Galunisertib inhibits glioma vasculogenic mimicry formation induced by astrocytes. *Sci. Rep.* **6**, 23056; doi: 10.1038/srep23056 (2016).
